# Clinical Spectrum and Outcomes of SOX1 Antibody‐Associated Paraneoplastic Neurological Syndromes: A Chinese Cohort Study

**DOI:** 10.1002/acn3.70313

**Published:** 2026-01-23

**Authors:** Jin‐Long Ye, Zu‐Ying Kuang, Bo Li, Meng‐Qiu Pan, Li‐Hua Zhou, Yang‐Yang Dai, Si‐Fen Xie, Xian‐Guang Lin, Ye‐Peng Hu, Li‐Hong Jiang, Zhan‐Hang Wang, Wei‐Jing Zhang

**Affiliations:** ^1^ Department of Neurology Guangdong Sanjiu Brain Hospital Guangzhou Guangdong China; ^2^ Department of Neurology, Institute of Neuroscience, Key Laboratory of Neurogenetics and Channelopathies of Guangdong Province and the Ministry of Education of China, The Second Affiliated Hospital Guangzhou Medical University Guangzhou Guangdong China

**Keywords:** Chinese population, clinical phenotype, immunotherapy, neuropsychiatric symptoms, paraneoplastic neurological syndrome, SOX1 antibody, treatment response

## Abstract

**Background:**

SOX1 antibody‐positive paraneoplastic neurological syndromes (PNS) exhibit significant population‐specific clinical heterogeneity. While Western cohorts predominantly manifest Lambert‐Eaton myasthenic syndrome (65%–80%), comprehensive clinical characterization and treatment response data in Asian populations remain critically limited.

**Methods:**

We conducted a single‐center retrospective case series analyzing 13 consecutive patients with SOX1 antibody‐positive PNS treated at Guangdong Sanjiu Brain Hospital from January 2019 to December 2024. SOX1 antibodies were confirmed using commercial immunoblot assay. Primary endpoints included treatment response (≥ 1‐point improvement on modified Rankin Scale [mRS]) and functional recovery (mRS ≤ 2). Statistical analyses employed Fisher's exact tests and Mann–Whitney *U* tests.

**Results:**

Among 13 patients (median age 61 years [IQR 56–67], 53.8% female), neuropsychiatric presentations predominated, including seizures (46.2%) and psychiatric symptoms (30.8%), with combined neuropsychiatric manifestations occurring in 53.8% of patients. Co‐existing neuronal antibodies were identified in 15.4% of cases (GABAB receptor, LGI1). Malignancy was confirmed in 30.8% of patients. Immunotherapy recipients (*n* = 7) demonstrated significantly superior functional outcomes compared to non‐treated patients: median 3‐month mRS 0 (IQR 0–0) versus 3 (IQR 3–3), *p* = 0.03. Treatment response rates were 85.7% versus 33.3% (*p* = 0.103).

**Conclusions:**

Chinese patients with SOX1 antibody‐positive PNS demonstrate a neuropsychiatric‐predominant phenotype (53.8%), contrasting markedly with Western cohorts. Early immunotherapy administration was associated with superior functional outcomes (median 3‐month mRS: 0 vs. 3, *p* = 0.03). These findings support comprehensive neuronal antibody profiling and early immunotherapy consideration in patients presenting with neuropsychiatric manifestations.

## 1 Introduction

Paraneoplastic neurological syndromes (PNS) constitute autoimmune disorders triggered by systemic malignancies [1, 2], mediated through molecular mimicry between onconeural antigens and neuronal proteins. Among established PNS biomarkers, SOX1 antibodies demonstrate high specificity for underlying tumors [3], particularly small‐cell lung carcinoma, being detected in approximately 65% of small‐cell lung cancer patients with concurrent neurological symptoms [4, 5].

Current epidemiological data from Western populations indicate that Lambert‐Eaton myasthenic syndrome (LEMS) represents the predominant phenotype in 65%–80% of SOX1‐positive cases [6], followed by cerebellar ataxia (15%–20%) and limbic encephalitis (10%–15%) [7]. SOX1 antibody‐positive patients typically demonstrate high tumor detection rates (> 90%), with small‐cell lung carcinoma accounting for the vast majority of associated malignancies [8, 9].

However, emerging evidence suggests potential population‐specific variations in PNS manifestations that may significantly impact diagnostic and therapeutic approaches. While multiple large‐scale studies have characterized SOX1‐related syndromes in Caucasian cohorts [10], comprehensive clinical data from Asian populations remain critically scarce. Preliminary case reports from Japanese and Korean centers suggest different phenotypic distributions, with neuropsychiatric presentations potentially being more prevalent than classic neuromuscular syndromes. This knowledge gap impedes optimized diagnosis and management of Asian patients presenting with neurological symptoms potentially linked to occult malignancies.

Furthermore, treatment response patterns in SOX1‐positive PNS remain poorly characterized across all populations. Limited evidence exists regarding optimal immunotherapy protocols, treatment timing, and outcome predictors [11]. The potential for antibody co‐existence and its clinical implications have been inadequately explored, particularly in non‐Western populations where autoimmune disease patterns may differ significantly [12].

To address these critical knowledge gaps, we conducted a comprehensive retrospective case series of Chinese patients with SOX1 antibody‐positive PNS, incorporating detailed neuronal antibody profiling and systematic outcome assessment. Our primary objectives were to: (1) characterize the clinical phenotype and spectrum in a Chinese population; (2) investigate co‐existing neuronal antibodies and their clinical significance; (3) evaluate immunotherapy response patterns and outcome predictors; and (4) identify population‐specific diagnostic and therapeutic considerations. This investigation aims to provide evidence‐based insights that could inform region‐specific clinical approaches while contributing to the global understanding of this rare but clinically significant autoimmune neurological disorder.

## 2 Methods

### 2.1 Study Design and Participants

This retrospective case series analyzed consecutive patients with SOX1 antibody‐positive paraneoplastic neurological syndromes admitted to Guangdong Sanjiu Brain Hospital from January 2019 to December 2024. The study protocol was approved by the Institutional Review Board of Guangdong Sanjiu Brain Hospital (Notification number: (2025) Medical No. (20)), and informed consent was waived given the retrospective nature using anonymized medical records.

Patients were included if they had: (1) confirmed SOX1 antibody positivity by commercial immunoblot assay; (2) age > 14 years; (3) neurological symptoms consistent with paraneoplastic syndrome; and (4) complete clinical data with ≥ 3‐month follow‐up. Exclusion criteria included concurrent systemic autoimmune diseases, incomplete clinical records, presence of dominant onconeural antibodies with higher titers than SOX1, established primary psychiatric or neurodegenerative diseases, and drug‐induced neurological syndromes.

### 2.2 SOX1 Antibody Detection and Additional Neuronal Antibody Screening

Serum SOX1 antibodies were detected using commercial immunoblot assay with recombinant SOX1 antigens. All 13 patients underwent comprehensive neuronal antibody screening including onconeural antibodies (Hu, Yo, Ri, CV2/CRMP5, Ma2, amphiphysin, Tr/DNER) using the same commercial immunoblot platform, and neuronal surface antibodies including NMDAR, LGI1, CASPR2, GABAB receptor, GABAA receptor, AMPAR, mGluR5, mGluR1, KLHL11, and glycine receptor antibodies using indirect immunofluorescence on transfected cells.

We acknowledge that voltage‐gated calcium channel P/Q‐type (VGCC‐PQ) antibodies were not tested in this cohort due to technical unavailability at our institution during the study period (2019–2024). This represents a limitation as SOX1 + VGCC‐PQ co‐existence is commonly reported in Western cohorts and may influence clinical phenotype, particularly Lambert‐Eaton myasthenic syndrome prevalence.

### 2.3 Clinical Assessment and Outcome Measures

Baseline characteristics included demographic data, presenting neurological symptoms, neuroimaging findings, and tumor status. Neurological function was assessed using the modified Rankin Scale (mRS) at baseline and follow‐up visits. Primary outcomes included treatment response (≥ 1‐point mRS improvement) and functional recovery (mRS ≤ 2). Secondary outcomes included time to response, tumor detection rate, and adverse events. Neurological symptoms were categorized as limb weakness, gait instability, seizures, cognitive impairment, psychiatric manifestations, cerebellar dysfunction, and dysarthria. Tumor evaluation included chest CT, abdominal imaging, and tumor markers (neuron‐specific enolase, carcinoembryonic antigen). Tumor status was determined through multidisciplinary evaluation incorporating imaging, histopathology, and biomarkers.

### 2.4 Treatment and Management

Immunotherapy was administered based on clinical judgment and included corticosteroids, intravenous immunoglobulin, and plasma exchange as clinically indicated. Treatment decisions were individualized according to symptom severity and patient condition. Specific immunotherapy regimens included: (1) high‐dose intravenous methylprednisolone (1000 mg daily for 3–5 days); (2) intravenous immunoglobulin (IVIG, 0.4 g/kg/day for 5 days); and (3) plasma exchange. Combined immunotherapy was administered in 3 patients, including 2 patients who received corticosteroids plus IVIG and 1 patient who received corticosteroids plus plasma exchange. Due to the retrospective nature of data collection, some treatment details (e.g., precise sequencing of combination therapies) were incompletely documented.

### 2.5 Statistical Analysis

Statistical analyses were primarily descriptive given the small sample size typical of rare disease studies. Continuous variables were presented as median with interquartile range (IQR), while categorical variables were presented as frequencies and percentages.

Between‐group comparisons used Fisher's exact test for categorical variables and Mann–Whitney *U* test for continuous variables. Multivariable analysis was not performed due to limited sample size. We acknowledge that the small sample size and baseline imbalances between treatment groups (including differences in baseline functional status, seizure prevalence, and phenotypic distribution) limit causal inference regarding treatment efficacy. These confounding factors could not be adjusted through multivariable analysis due to sample size constraints. Statistical significance was set at *p* < 0.05. All analyses were performed using R software version 4.3.0.

## 3 Results

### 3.1 Demographic and Clinical Characteristics

Between January 2019 and December 2024, 13 consecutive patients with confirmed SOX1 antibody‐positive paraneoplastic neurological syndromes were identified and included in the analysis. The cohort comprised 7 females (53.8%) and 6 males (46.2%) with a median age of 61 years (interquartile range [IQR] 56–67 years, range 34–78 years).

Neurological presentations demonstrated marked heterogeneity. The most frequent manifestations included seizures in 6 patients (46.2%), limb weakness in 6 patients (46.2%), ataxia in 4 patients (30.8%), and psychiatric symptoms in 4 patients (30.8%). Notably, combined neuropsychiatric presentations (seizures and/or psychiatric symptoms) occurred in 7 patients (53.8%), representing the predominant phenotypic pattern in this cohort. Additional manifestations included gait instability in 4 patients (30.8%) and cognitive impairment in 3 patients (23.1%). Detailed baseline characteristics and symptom distribution between treatment groups are summarized in Table 1.

**TABLE 1:** Baseline characteristics of SOX1 antibody‐positive patients.

Characteristics	All (*n* = 13)	Immunotherapy (*n* = 7)	Non‐immunotherapy (*n* = 6)	*p*
Male, *n* (%)	6 (46.2)	3 (42.9)	3 (50.0)	1.00
Female, *n* (%)	7 (53.8)	4 (57.1)	3 (50.0)	1.00
Age, median (IQR), y	61 (56–67)	61 (46–63)	64 (58–67)	0.28
Seizures, *n* (%)	6 (46.2)	5 (71.4)	1 (16.7)	0.10
Ataxia, *n* (%)	4 (30.8)	1 (14.3)	3 (50.0)	0.27
Limb weakness, *n* (%)	6 (46.2)	2 (28.6)	4 (66.7)	0.29
Gait instability, *n* (%)	4 (30.8)	1 (14.3)	3 (50.0)	0.27
Psychiatric symptoms, *n* (%)	4 (30.8)	4 (57.1)	0 (0.0)	0.07
Confirmed tumor, *n* (%)	4 (30.8)	2 (28.6)	2 (33.3)	1.00
Baseline mRS, median (IQR)	3 (1–3)	2 (1–2)	3 (3–3)	0.09
3‐mo mRS, median (IQR)	1 (0–3)	0 (0–0)	3 (3–3)	0.03*

*Note:* Data are presented as median (IQR) for continuous variables and *n* (%) for categorical variables. *p*‐Values from Fisher's exact test for categorical variables and Mann–Whitney *U* test for continuous variables.

* *p* < 0.05 (Mann–Whitney *U* test or Fisher's exact test as appropriate).

### 3.2 Malignancy Detection and Tumor Characteristics

Confirmed malignancy was identified in 4 patients (30.8% tumor detection rate). All identified tumors were small‐cell lung carcinomas, diagnosed through histopathological examination following chest imaging abnormalities. The remaining 9 patients (69.2%) showed no evidence of underlying malignancy despite comprehensive screening including chest and abdominal computed tomography, positron emission tomography‐computed tomography when indicated, and serial tumor marker assessments. Among the 9 initially tumor‐negative patients, extended surveillance was conducted during follow‐up. One patient developed radiologically confirmed small‐cell lung carcinoma at 18‐month follow‐up. The cumulative tumor detection rate was 38.5% (5/13) at 24 months compared to 30.8% (4/13) at initial screening.

### 3.3 Neuronal Antibody Co‐Existence Pattern

Comprehensive neuronal antibody profiling revealed co‐existing antibodies in 2 patients (15.4%). One patient demonstrated concurrent GABAB receptor antibodies alongside SOX1 positivity, presenting with refractory seizures and cognitive decline. The second patient exhibited co‐existing LGI1 antibodies with SOX1, manifesting as psychiatric symptoms and memory impairment. Both patients with antibody co‐existence received immunotherapy and achieved complete functional recovery (mRS = 0 at 3‐month follow‐up).

### 3.4 Treatment Administration and Protocols

Seven patients (53.8%) received immunotherapy, while 6 patients (46.2%) were managed conservatively due to mild symptoms (*n* = 3), patient preference (*n* = 2), or medical contraindications (*n* = 1). Among treated patients, therapeutic protocols included: high‐dose intravenous methylprednisolone in 5 patients (71.4%), intravenous immunoglobulin in 4 patients (57.1%), and therapeutic plasmapheresis in 2 patients (28.6%). Three patients (42.9%) received combination therapy. The median time from symptom onset to treatment initiation was 28 days (IQR 14–45 days).

### 3.5 Primary Treatment Outcomes

Functional outcomes demonstrated significant differences between treatment groups at 3‐month follow‐up. The immunotherapy group achieved a median mRS score of 0 (IQR 0–0) compared to 3 (IQR 3–3) in the non‐immunotherapy group (*p* = 0.03, Mann–Whitney *U* test).

**FIGURE 1:**
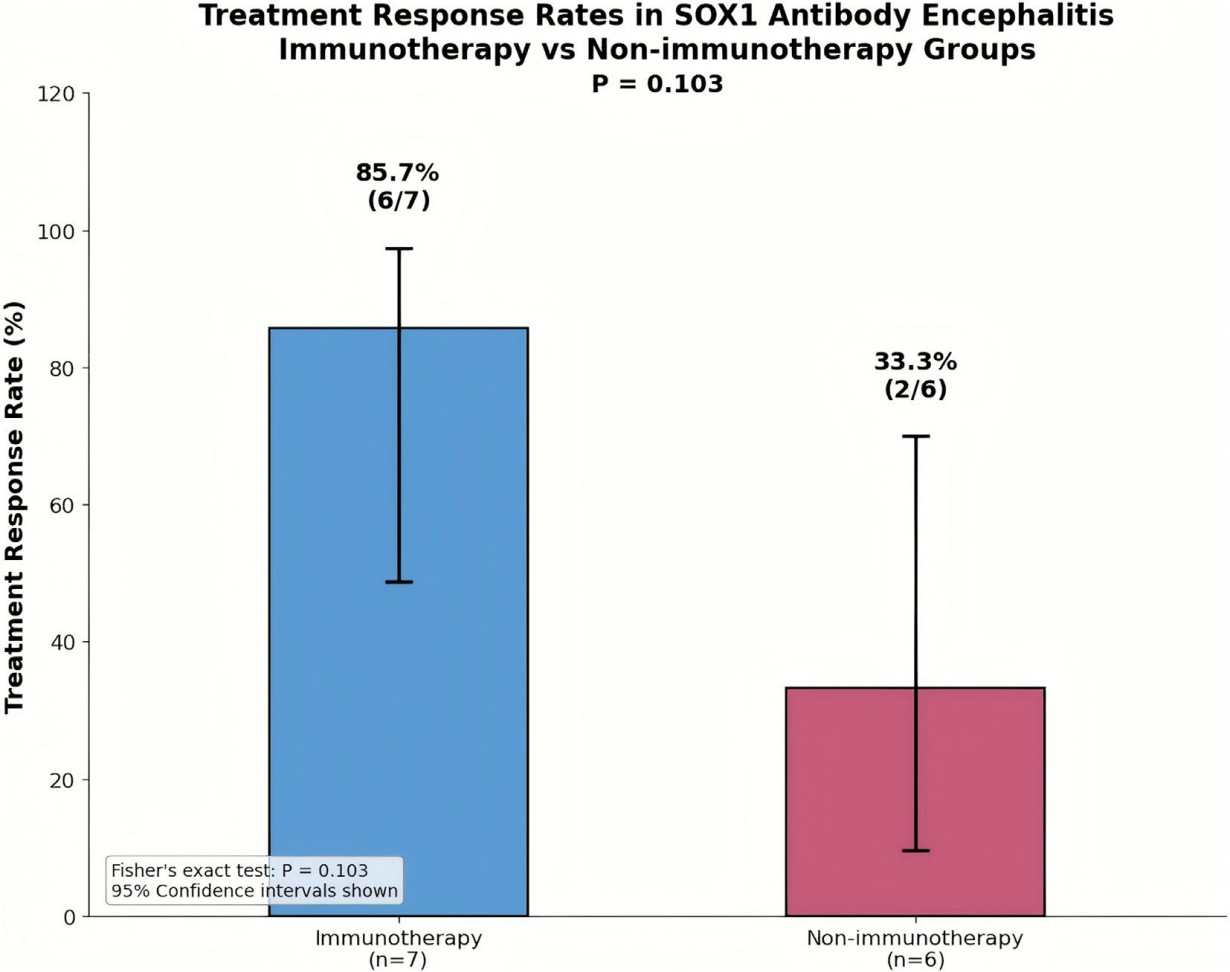
Treatment response rates in SOX1 antibody‐associated encephalitis patients by treatment group. Treatment response rates were numerically higher in the immunotherapy group compared to the non‐immunotherapy group (85.7% [6/7] vs 33.3% [2/6], *P* = 0.103, Fisher's exact test), though statistical significance was not achieved.

Treatment response, defined as ≥ 1‐point improvement in mRS score, was observed in 8 patients (61.5%) overall. Response rates were markedly higher in the immunotherapy group (6/7 patients, 85.7%) compared to the conservative management group (2/6 patients, 33.3%; *p* = 0.103, Fisher's exact test) (Figure 1). Complete functional recovery (mRS ≤ 1) was achieved by 7 patients (53.8%) overall, with significant differences favoring immunotherapy recipients (6/7 patients, 85.7% vs. 1/6 patients, 16.7%; *p* = 0.025, Fisher's exact test). Comprehensive treatment outcomes and functional assessments are presented in Table 2.

**TABLE 2:** Treatment outcomes and functional recovery.

Outcome measure	All patients (*n* = 13)	Immunotherapy (*n* = 7)	Non‐immunotherapy (*n* = 6)	*p*
*3‐Month outcomes*				
mRS score, median (IQR)	1 (0–3)	0 (0–0)	3 (3–3)	0.03*
Treatment response (≥ 1‐point mRS improvement), *n* (%)	8 (61.5)	6 (85.7)	2 (33.3)	0.103
Complete recovery (mRS ≤ 1), *n* (%)	7 (53.8)	6 (85.7)	1 (16.7)	0.025*
*Overall prognosis distribution, n (%)*				
Improved	8 (61.5)	6 (85.7)	2 (33.3)	—
Stable	5 (38.5)	1 (14.3)	4 (66.7)	—

* *p* < 0.05.

### 3.6 Secondary Outcomes and Long‐Term Follow‐Up

Median follow‐up duration was 8 months (IQR 5–14 months, range 3–24 months). No treatment‐related serious adverse events were documented. Among immunotherapy recipients, symptom improvement was typically observed within 2–4 weeks of treatment initiation. Disease relapse occurred in 1 patient (7.7%) who initially responded to treatment but experienced symptom recurrence at 6 months, successfully managed with repeated immunotherapy.

### 3.7 Clinical Phenotype‐Outcome Relationships

Seizures demonstrated the strongest association with favorable outcomes, with all 6 patients with seizures achieving treatment response compared to 2/7 patients without seizures (*p* = 0.011). Combined neuropsychiatric presentations (seizures and/or psychiatric symptoms, *n* = 7) showed superior outcomes compared to those without neuropsychiatric features (*n* = 6), with 6/7 patients (85.7%) achieving complete recovery versus 1/6 patients (16.7%; *p* = 0.025). Conversely, ataxia showed trends toward poorer outcomes, though not statistically significant (*p* = 0.127).

Analysis of phenotype‐outcome associations revealed distinct patterns. Patients presenting with neuropsychiatric‐dominant symptoms demonstrated superior treatment responses compared to those with motor‐dominant symptoms. Specifically, among 7 patients with neuropsychiatric presentations, 6 (85.7%) achieved complete recovery compared to 1/6 patients (16.7%) with primarily motor symptoms (*p* = 0.025).

Seizures as a presenting feature were associated with favorable outcomes across all patients (6/6 patients with seizures achieved treatment response vs. 2/7 patients without seizures; *p* = 0.011). Conversely, ataxia was more commonly observed in patients with poor outcomes, though statistical significance was not achieved due to small sample size (1/4 patients with ataxia achieved good outcomes vs. 7/9 patients without ataxia; *p* = 0.127). Detailed clinical phenotype and outcome associations are shown in Table 3.

**TABLE 3:** Clinical characteristics associated with treatment response.

Characteristics	Good outcomes (*n* = 8)	Poor outcomes (*n* = 5)	*p*	OR (95% CI)
*Neurological manifestations*				
Epileptic seizures, *n* (%)	6 (75.0)	0 (0.0)	0.011*	NA
Ataxia, *n* (%)	1 (12.5)	3 (60.0)	0.127	0.11 (0.01–1.32)
Limb weakness, *n* (%)	3 (37.5)	3 (60.0)	0.608	0.40 (0.05–3.20)
*Treatment and disease factors*				
Immunotherapy received, *n* (%)	6 (75.0)	1 (20.0)	0.103	12.0 (0.96–150.0)
Confirmed malignancy, *n* (%)	2 (25.0)	2 (40.0)	0.608	0.50 (0.06–4.35)
Age ≤ 60 years, *n* (%)	4 (50.0)	1 (20.0)	0.336	4.0 (0.35–45.8)

*Note:* Fisher's exact test.

Abbreviations: CI, confidence interval; NA, not applicable due to zero cells; OR, odds ratio.

* *p* < 0.05.

### 3.8 Predictors of Treatment Response

Univariate analysis identified several factors associated with favorable outcomes. Receipt of immunotherapy emerged as the strongest predictor of good functional outcome (odds ratio 12.0, 95% confidence interval 0.96–150.0; *p* = 0.103). Neuropsychiatric presentations, particularly seizures, were significantly associated with treatment responsiveness. Age, sex, and presence of underlying malignancy showed no significant association with treatment outcomes (all *p* > 0.05).

Notably, tumor detection status did not influence treatment response patterns, with similar improvement rates observed between patients with confirmed malignancy (2/4 patients, 50%) and those without identifiable tumors (6/9 patients, 66.7%; *p* = 0.608). This finding supports the hypothesis that neurological symptoms are primarily mediated by antibody‐related autoimmune mechanisms rather than direct tumor effects.

## 4 Discussion

### 4.1 Principal Findings and Clinical Significance

This retrospective case series provides the first comprehensive characterization of SOX1 antibody‐positive paraneoplastic neurological syndromes in a Chinese population, revealing distinct clinical phenotypes and treatment response patterns that differ substantially from previously reported Western cohorts [13]. Our most significant finding is the predominance of neuropsychiatric presentations (53.8%) in Chinese patients, contrasting sharply with Western populations where Lambert‐Eaton myasthenic syndrome accounts for 65%–80% of cases. This neuropsychiatric‐predominant phenotype, characterized by seizures (46.2%) and psychiatric symptoms (30.8%), represents a novel clinical pattern that may reflect population‐specific genetic, immunological, or environmental factors influencing autoimmune neurological disease expression.

The favorable outcomes in immunotherapy recipients (85.7% response rate) suggest potential benefits of early intervention, though baseline confounders limit causal inference. More importantly, the significant functional outcome differences between treated and untreated groups (median 3‐month mRS: 0 vs. 3, *p* = 0.03) demonstrate clinically meaningful benefits that justify immunosuppressive therapy even in the absence of identified malignancy. This finding challenges the traditional approach of withholding immunotherapy pending tumor identification and suggests that neurological symptom severity, rather than malignancy status, should guide treatment decisions in SOX1‐positive patients.

### 4.2 Population‐Specific Phenotypic Variations and Mechanistic Implications

The striking phenotypic differences between our Chinese cohort and Western populations warrant detailed consideration of underlying mechanisms. While Western studies consistently report neuromuscular manifestations as the dominant phenotype, our findings suggest that SOX1 antibodies may target different neuroanatomical regions or express distinct epitope specificities in Asian populations. The high prevalence of seizures and psychiatric symptoms in our cohort indicates preferential involvement of limbic and cortical structures, potentially reflecting genetic variations in SOX1 expression patterns, major histocompatibility complex associations, or differential immune response profiles across ethnic groups.

This phenotypic divergence has profound diagnostic implications, as clinicians in Asia should maintain high suspicion for SOX1‐related syndromes in patients presenting with neuropsychiatric symptoms, even without classic neuromuscular features. The diagnostic challenges encountered with Lambert‐Eaton myasthenic syndrome in our cohort suggest that existing diagnostic algorithms, predominantly derived from Western populations, may require adaptation for Asian clinical settings, emphasizing the need for region‐specific diagnostic strategies and enhanced clinical awareness programs.

### 4.3 Antibody Co‐Existence Patterns and Clinical Implications

Our identification of co‐existing neuronal antibodies in 15.4% of patients represents a higher prevalence than previously reported in SOX1‐positive cohorts (typically < 5%) [14]. This finding has several important implications. First, it suggests that comprehensive neuronal antibody profiling is essential in SOX1‐positive patients, as single‐antibody testing may miss clinically relevant co‐existing antibodies that could influence both phenotype and treatment responsiveness. Second, the excellent treatment outcomes achieved by both patients with antibody co‐existence (both achieved mRS = 0) indicate that multiple antibody positivity does not necessarily confer treatment resistance, contrary to some reports in other autoimmune encephalitis contexts [15].

The specific combination of SOX1 with GABAB receptor and LGI1 antibodies observed in our series is particularly noteworthy, as both co‐existing antibodies are associated with seizure disorders and psychiatric manifestations [16]. This pattern suggests potential mechanistic synergy between different antibody targets in producing the neuropsychiatric phenotype predominant in our cohort, supporting the hypothesis that antibody co‐existence may contribute to the distinct clinical presentation observed in Asian populations.

Our antibody co‐existence pattern (SOX1 with GABAB receptor and LGI1) differs from Western cohorts where SOX1 + VGCC‐PQ predominates. This divergence may reflect: (1) undetected VGCC‐PQ antibodies in our cohort due to testing limitations; (2) genuine population‐specific immunological differences; or (3) referral bias at our center, which specializes in autoimmune encephalitis and may preferentially receive neuropsychiatric presentations. Future studies with comprehensive antibody panels including VGCC‐PQ testing are needed to clarify these geographic patterns.

### 4.4 Malignancy Detection Rates and Screening Implications

The relatively low malignancy detection rate in our cohort (30.8%) compared to classic onconeural antibodies (typically 60%–90%) [17] raises important questions about optimal cancer screening strategies in SOX1‐positive patients. While all identified tumors were small‐cell lung carcinomas, confirming the established association, the majority of patients (69.2%) showed no evidence of underlying malignancy despite comprehensive screening protocols.

The phenotypic divergence observed between tumor‐positive and tumor‐negative patients in our cohort provides additional insights into this discrepancy. Tumor‐positive patients predominantly manifested motor phenotypes (3/4 patients, 75%), whereas tumor‐negative patients more frequently presented with neuropsychiatric symptoms (6/9 patients, 67%). This pattern suggests potential mechanistic heterogeneity, where neuropsychiatric presentations may reflect more autoimmune‐predominant mechanisms with lower malignancy association, while motor phenotypes may have stronger paraneoplastic linkage.

Furthermore, our relatively short median follow‐up duration (8 months) and the absence of standardized long‐term surveillance protocols mean that many “tumor‐negative” patients may represent “tumor‐not‐yet‐detected” cases rather than confirmed absence of malignancy. The undetected presence of VGCC‐PQ antibodies in our cohort (which we did not test) may also contribute to the lower tumor detection rate, as SOX1 + VGCC‐PQ combinations show particularly high malignancy rates in Western literature. Despite these considerations, our findings underscore that SOX1 antibodies should continue to be regarded as high‐risk paraneoplastic markers warranting comprehensive and prolonged tumor surveillance.

This finding suggests that SOX1 antibodies may represent a distinct category of onconeural antibodies with different tumor association patterns compared to traditional intracellular antibodies such as Hu, Yo, or Ri [18]. The lower tumor detection rates may reflect: (1) earlier detection of neurological symptoms before the tumor becomes radiologically apparent; (2) different tumor biology with slower growth patterns; (3) potential non‐paraneoplastic autoimmune mechanisms in certain patients; or (4) population‐specific differences in cancer epidemiology and SOX1 antibody expression.

These observations have practical implications for clinical management, suggesting that extended surveillance intervals or alternative screening modalities may be warranted in SOX1‐positive patients without initial evidence of malignancy. However, the consistent association with small‐cell lung carcinoma when tumors are identified reinforces the importance of thorough pulmonary evaluation and smoking cessation counseling in all SOX1‐positive patients.

### 4.5 Treatment Response Predictors and Clinical Decision‐Making

Our analysis identified several key factors associated with favorable treatment outcomes that can inform clinical decision‐making. The strong association between neuropsychiatric presentations and treatment responsiveness (85.7% response rate in neuropsychiatric phenotype vs. 16.7% in motor‐predominant phenotype) suggests that symptom pattern may serve as a valuable predictor of immunotherapy benefit. Specifically, the presence of seizures emerged as the strongest individual predictor of treatment success, with all seizure patients achieving favorable outcomes compared to only 28.6% of non‐seizure patients.

It is important to acknowledge that the observed association between immunotherapy and favorable outcomes must be interpreted with caution due to significant baseline confounders. As noted in our statistical analysis, patients receiving immunotherapy had better baseline functional status (median mRS 2 vs. 3), higher prevalence of seizures (71.4% vs. 16.7%), and predominantly neuropsychiatric phenotypes (85.7% vs. 16.7%) compared to the non‐immunotherapy group. When outcomes are examined by phenotype rather than treatment status, a clear pattern emerges: all 7 patients with neuropsychiatric presentations achieved excellent outcomes (6/7 with mRS ≤ 1) regardless of treatment modality, while patients with motor‐predominant phenotypes showed poor outcomes (1/6 with mRS ≤ 1) irrespective of immunotherapy administration.

This phenotype‐driven outcome pattern suggests that clinical presentation—rather than treatment modality—may be the primary determinant of prognosis in SOX1‐positive PNS. The superior outcomes in neuropsychiatric phenotypes may reflect greater reversibility of cortical and limbic inflammation compared to brainstem or cerebellar motor circuit degeneration, or the presence of co‐existing surface antibodies (GABAB receptor, LGI1) that are more amenable to immunotherapy.

Several mechanisms may further explain this differential treatment responsiveness. First, both patients with neuropsychiatric presentations in our cohort had co‐existing surface antibodies (GABABR, LGI1), which mediate reversible receptor internalization rather than irreversible cytotoxic T‐cell damage associated with intracellular onconeural antibodies. Second, seizures and psychiatric symptoms primarily reflect neuronal hyperexcitability and synaptic dysfunction, pathophysiological processes that are more amenable to immunomodulation than structural damage to cerebellar Purkinje cells or neuromuscular junctions seen in motor phenotypes. Third, the acute nature of seizure presentations may enable earlier medical attention and treatment initiation before irreversible neuronal damage accumulates.

Therefore, while our data support immunotherapy consideration in SOX1‐positive patients, definitive establishment of treatment efficacy requires prospective studies with balanced baseline characteristics or adequately powered trials enabling multivariable adjustment for confounders.

Conversely, the tendency for poor outcomes in patients with ataxia, though not statistically significant due to sample size limitations, aligns with previous reports indicating that cerebellar involvement in autoimmune disorders often shows limited treatment responsiveness [19]. This pattern may reflect the vulnerability of cerebellar Purkinje cells to immune‐mediated damage or the difficulty in achieving adequate immunosuppressive drug penetration into cerebellar tissue.

The emergence of immunotherapy as the strongest overall outcome predictor (OR 12.0, *p* = 0.103) suggests potential benefits of early treatment initiation, though statistical significance was not achieved. The median time from symptom onset to treatment (28 days) in our cohort suggests potential for further improvement in outcomes through enhanced clinical recognition and accelerated diagnostic pathways.

### 4.6 Clinical Implications and Future Directions

Our findings have several immediate clinical implications. First, they support the development of population‐specific diagnostic criteria for SOX1‐related syndromes, particularly in Asian populations where neuropsychiatric presentations may predominate. Second, they provide evidence‐based support for early immunotherapy initiation in SOX1‐positive patients, regardless of malignancy detection status, based on the significant functional benefits observed.

Future research priorities should include: (1) larger multicenter Asian cohorts to validate our phenotypic observations; (2) comparative immunogenetic studies to identify population‐specific factors influencing disease expression; (3) prospective treatment trials optimizing immunotherapy protocols for different phenotypic presentations; (4) longitudinal studies examining long‐term outcomes and relapse patterns; and (5) investigation of biomarkers predicting treatment responsiveness to guide personalized therapy approaches.

### 4.7 Study Limitations and Methodological Considerations

Several limitations must be acknowledged in interpreting our findings. The retrospective design introduces potential selection and information bias, while the single‐center recruitment may limit generalizability across different healthcare settings and geographic regions within China. The modest sample size (*n* = 13), while comparable to other rare disease studies, limits statistical power for detecting smaller effect sizes and precludes multivariable analysis of outcome predictors.

Technical limitations include the reliance on immunoblot‐based SOX1 detection without cell‐based assay confirmation, which may result in false‐positive results or miss low‐titer antibodies with clinical significance. Most notably, the absence of VGCC‐PQ antibody testing represents a significant limitation, as SOX1 + VGCC‐PQ is the most frequently reported antibody combination in Western cohorts and strongly correlates with Lambert‐Eaton myasthenic syndrome and small‐cell lung cancer. This testing gap may have led to underestimation of antibody co‐existence rates and influenced our phenotypic observations, particularly the lower LEMS prevalence in our cohort. The variable follow‐up duration (3–24 months) may affect outcome assessment consistency, particularly for capturing late treatment responses or disease relapses. Additionally, the absence of standardized treatment protocols, while reflecting real‐world clinical practice, introduces treatment variability that may confound outcome comparisons.

Despite these limitations, our study provides valuable insights into a rare but clinically important condition, filling a critical knowledge gap regarding SOX1‐related syndromes in Asian populations. The findings warrant validation in larger, prospective multicenter studies but offer immediate clinical guidance for managing SOX1‐positive patients in Asian healthcare settings.

## 5 Conclusion

This study reveals important population‐specific differences in SOX1‐associated paraneoplastic neurological syndromes between Chinese and Western populations. The predominant neuropsychiatric phenotype and favorable immunotherapy response patterns observed in Chinese patients have significant implications for diagnosis and treatment approaches. These findings warrant validation in larger prospective multicenter studies to establish evidence‐based treatment guidelines for Asian populations with SOX1‐positive paraneoplastic neurological syndromes.

## Author Contributions

**Jin‐Long Ye:** methodology, data curation, formal analysis, investigation, writing – original draft. **Zu‐Ying Kuang:** methodology, data curation, investigation. **Bo Li:** data curation, investigation. **Meng‐Qiu Pan:** data curation, investigation. **Li‐Hua Zhou:** data curation. **Yang‐Yang Dai:** data curation. **Si‐Fen Xie:** data curation. **Xian‐Guang Lin:** data curation. **Ye‐Peng Hu:** data curation. **Li‐Hong Jiang:** data curation. **Zhan‐Hang Wang:** conceptualization, writing – review and editing, supervision, project administration. **Wei‐Jing Zhang:** conceptualization, methodology, formal analysis, writing – original draft, writing – review and editing, supervision. All authors have read and approved the final manuscript.

## Funding

The authors have nothing to report.

## Conflicts of Interest

The authors declare no conflicts of interest.

## Data Availability

The anonymized data supporting the findings of this study are not publicly available due to patient privacy and ethical restrictions but are available from the corresponding authors upon reasonable request with appropriate ethical approval. Data sharing is subject to approval by the Institutional Review Board of Guangdong Sanjiu Brain Hospital and compliance with patient privacy regulations.
